# Hypertrophic Cardiomyopathy: From Phenotype and Pathogenesis to Treatment

**DOI:** 10.3389/fcvm.2021.722340

**Published:** 2021-10-25

**Authors:** Zeyi Cheng, Tingting Fang, Jinglei Huang, Yingqiang Guo, Mahboob Alam, Hong Qian

**Affiliations:** ^1^Department of Cardiovascular Surgery, West China Hospital, Sichuan University, Chengdu, China; ^2^Department of Cardiology, West China Hospital, Sichuan University, Chengdu, China; ^3^School of Medicine, Lanzhou University, Lanzhou, China; ^4^Division of Cardiovascular Medicine, Department of Medicine, Baylor College of Medicine, Houston, TX, United States

**Keywords:** hypertrophic cardiomyopathy, phenotype, pathogenesis, treatment, review

## Abstract

Hypertrophic cardiomyopathy (HCM) is a very common inherited cardiovascular disease (CAD) and the incidence is about 1/500 of the common population. It is caused by more than 1,400 mutations in 11 or more genes encoding the proteins of the cardiac sarcomere. HCM presents a heterogeneous clinical profile and complex pathophysiology and HCM is the most important cause of sudden cardiac death (SCD) in young people. HCM also contributes to functional disability from heart failure and stroke (caused by atrial fibrillation). Current treatments for HCM (medication, myectomy, and alcohol septal ablation) are geared toward slowing down the disease progression and symptom relief and implanted cardiac defibrillator (ICD) to prevent SCD. HCM is, however, entering a period of tight translational research that holds promise for the major advances in disease-specific therapy. Main insights into the genetic landscape of HCM have improved our understanding of molecular pathogenesis and pointed the potential targets for the development of therapeutic agents. We reviewed the critical discoveries about the treatments, mechanism of HCM, and their implications for future research.

## Introduction

**Figure 1:**
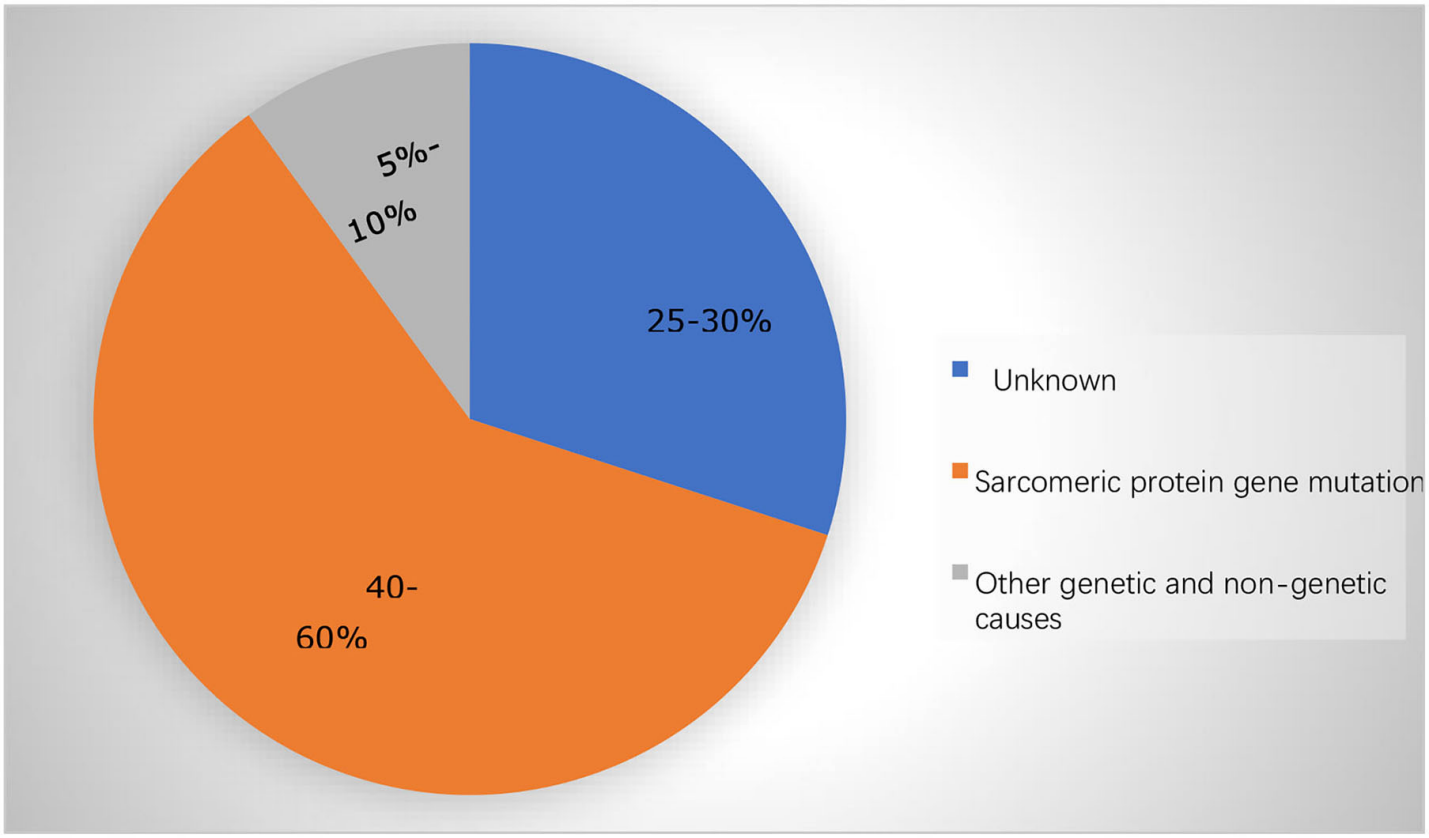
The majority of the cases in adolescents and adults are caused by mutations in the sarcomere protein genes.

Hypertrophic cardiomyopathy (HCM) is a heterogeneous myocardial disease characterized by left ventricular hypertrophy and in the cases of hypertrophic obstructive cardiomyopathy (HOCM), it is characterized by asymmetric septal hypertrophy. In the majority of the cases, the average interventricular septal thickness is 26 mm, which cannot be fully explained by the loading conditions of the left ventricle (1, 2). There are several types of HCM based on the distribution of hypertrophy: symmetric, asymmetric, apical, and focal (3). In addition to the hypertrophy, the abnormalities of the mitral valve and subvalvular apparatus lead to the systolic anterior motion (SAM) and left ventricular outflow tract (LVOT) obstruction in about two-thirds of the HCM cases, the characteristic features of HOCM, as well as the microvascular dysfunction and subendocardial ischemia (3). Due to a combination of these factors, patients with HCM frequently experience reduced exercise capacity, dyspnea, and/or chest pain. HCM is mainly inherited in an autosomal dominant pattern, linked with mutations (nucleotide sequence variants) in 11 or more genes encoding the proteins of myocardial sarcomere structure (~60% of all the causes and >90% of the genetically defined patients), and with beta-myosin heavy chain and myosin-binding protein C genes most commonly involved (Table 1; Figure 1) (4–6). Patients with HCM suffer from the cardiovascular death rates of 1-2% per year including the sudden cardiac death (SCD) ~ 1%, heart failure (HF) ~ 0.5%, and thromboembolism ~ 0.1% (2, 3). In recent years, there has been tremendous development in this field with a translation of the basic science discoveries into the new therapeutic methods. In this study, we reviewed the recent development in pharmacological and gene-based therapies, which we believe will result in a comprehensive understanding of the treatment of HCM in the future.

**Table 1:** A list of the genes in which the pathogenic mutations are associated with hypertrophic cardiomyopathy (HCM).

**Gene**	**HCM frequence**	**Protein or associated phenotypes**
**Sarcomeric proteins**		
MYH7	40-44%	β-myosin heavy chain 7
MYBPC3	35-40%	Myosin-binding protein C3
TNNT2	5-15%	Troponin T
TNNI3	5%	Troponin I
TPM1	3%	Tropomyosin α-1 chian
MYL2	1-2%	Regulatory myosin light chain
MYL3	1%	Essential myosin light chain
ACTC1	1%	Actin
TNNC1	<1%	Troponin C
**Z-disk proteins**		
LBD3	1-5%	ZASP-LIM binding domain 3
ACTN2	<1%	Alpha-Actinin-2
ANKRD1	<1%	Ankyrin repeat domain-containing protein-1
CSRP3	<1%	Muscle LIM Protein
MYOZ2	<1%	Myozenin-2
TCAP	<1%	Telethonin
VCL	<1%	Vinculin
NEXT	<1%	Nexilin
FLNC	<1%	Filamin C
**Sarcomere-associated proteins**		
DES	<1%	Desmin

*It also includes the proteins and related phenotypes that are involved in these specific gene mutations and their proportion in the overall HCM*.

## Pathogenesis

**Figure 2:**
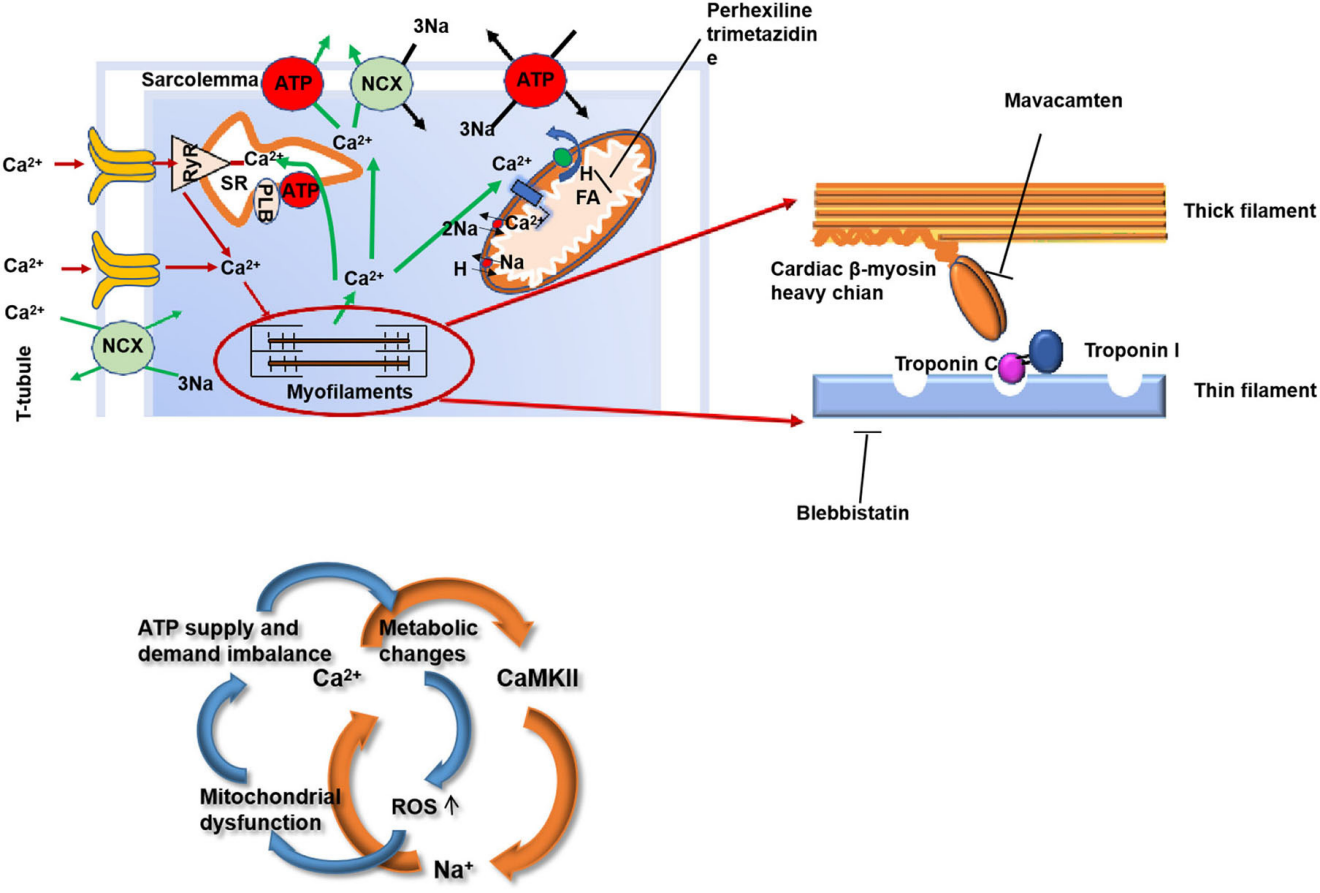
The structure of the myocardium and the mechanism of myocardial contraction are the potential targets for HCM therapy. HCM, hypertrophic cardiomyopathy; RyR, ryanodine receptor; SR, sarcoplasmic reticulum; NCX, sodium/calcium exchange pump; PLB, phospholamban; ROS, reactive oxygen species; FA, fatty acid.

Gene mutation is the initiating pathogenesis of HCM affecting the proteins by playing a critical role in the function of the cardiac muscle unit “sarcomeres.” The function of the sarcomere may weaken due to an abnormality in or shortage of any one of these proteins, which, in turn, affects the normal myocardial contractility. It is still not exactly described how the mutations in the sarcomere-related genes cause hypertrophy of the heart muscle (7, 8). However, there are several hypotheses that are as follows (Figure 2):

1. Mutations in the sarcomere-related genes are associated with an increased affinity for calcium in the myofilaments, activate the calmodulin kinase II (CaMKII) pathway, and delay the downstream targets of the CaMKII sodium channels, thus increase the intracellular calcium and, thus, forming a vicious cycle (9–12). This results in the impaired relaxation and diastolic dysfunction of the myocardium.
2. Mutations in the sarcomere-related genes in HCM can lead to inefficient contractility with a resultant increase in the ATP demand. This impairs the structure and function of the mitochondria leading to energy supply disorders (13–16). Microvascular dysfunction further exacerbates the myocardial energy deficiency of HCM and restricts the transport of the oxidative metabolites. The imbalance between the energy supply and demand leads to the myocardial cells in a state of peroxidation and then produces various reactive oxygen species (ROS), resulting in the glutathione acylation of the muscle filaments [cardiac myosin-binding protein C (cMyBP-C)] (17, 18). Functionally, this modification increases the myofilament calcium sensitivity and inhibits the kinetics of cross-bridge cycling, leading to the diastolic dysfunction and ultimately aggravating the HCM phenotype (18–21).
3. Due to the mutations in the sarcomere-related genes, the accumulation of the harmful proteins results in a toxic effect on the myocardial contractile devices and myocardial cells (22).
4. Sarcomeric protein transcription and posttranslational modifications, as well as the other modified genes, also promote the development of HCM. Studies have shown that polymorphism of angiotensin I can contribute to the hypertrophic phenotype (23). These modified factors stimulate non-cardiac cell proliferation such as fibroblasts (23), thereby promoting the development of HCM. In conclusion, the functional changes at the cellular and molecular levels could be target of innovative therapies.

The structural abnormalities of HCM include the following:

1. Abnormal myofibrils and abnormal arrangement of the cardiomyocytes (24).
2. Coronary artery microvascular dysfunction. Thickening of the blood vessel wall leading to asymptomatic myocardial ischemia, further inducing myocardial injury and fibrosis (25, 26). Interstitial connective tissue increases significantly. Fibrosis is patchy or widely distributed around the cells and poor remodeling of the myocardial tissue ultimately leads to irreversible dysfunction such as severe HF and SCD (27, 28).

## Novel Therapies

### Calcium Desensitizer

Ca^2+^ overload, CaMKII, and increased I_NaL_ play a very important role that drive the myocardial remodeling from the earliest stage of the development of hypertrophy, diastolic dysfunction, and the arrhythmogenic substrate (5, 6). There are many studies aimed at an increased calcium sensitivity (Figure 2).

### Blebbistatin

Blebbistatin is an inhibitor of actin-myosin interaction functioning independently of Ca^2+^ influx (29). Studies have shown that blebbistatin, in a mouse model of HCM caused by troponin T mutation, can reduce the sensitivity of Ca^2+^ to myofilaments and the incidence of arrhythmias; meanwhile, several studies Grillo et al. also reported that reducing the sensitivity of Ca^2+^ to myofilaments can be a target for the HCM treatment (29–32).

### Parvalbumin

Parvalbumin is a Ca^2+^ buffering molecule not expressed in the cardiomyocytes; when the concentration of Ca^2+^ increases, parvalbumin will release Mg^2+^ and binds to Ca^2+^. Piguet et al. and Coutu et al. found that parvalbumin can correct the myocardial diastolic dysfunction in the rat and mouse HCM models (33, 34).

### SERCA2a

In a mouse model of HCM caused by a tropomyosin mutation, SERCA2a, an SR protein, was transported by the adenovirus to 1-day-old mice. After several weeks, The sarco/endoplasmic reticulum calcium ATPase 2a isoform (SERCA2a) protein expression increased in the heart of the mouse and significantly improved the morphology of the heart. In HCM mice knocked out with SERCA2a inhibitory protein, phosphoprotein gene [phospholamban (PLN)] can also increase the absorption of Ca^2+^ by the sarcoplasmic reticulum (SR) and improve the phenotype (35).

### Diltiazem

Diltiazem can inhibit the L-type calcium channels (34). Early application of diltiazem caused the upregulation of the SR protein and eased the development of the HCM phenotype (36). In recent years, a study by Ho CY et al. found that diltiazem may relieve left ventricular remodeling in the asymptomatic sarcomere mutation carriers (NCT00319982) (37). The summarized novel therapies can be seen in Table 2.

**Table 2:** Table for the novel therapies.

**Novel therapies**	**Targets**	**Mechanisms**
Blebbistatin	Troponin T mutation	sensitivityofCa2+↓
Parvalbumin	Ca^2+^	Decrease the concentration of Ca2+
SERCA2a	Ca^2+^	SERCA2 a protein expression increased
Diltiazem	L-type calcium channels	Upregulation of the SR protein

## Metabolic Regulation-Energy Expenditure Hypothesis

In HCM, the mutations in the sarcomere gene result in reduced contractile efficiency of the sarcomere and an increase in ATP consumption. The characteristic of the HCM substrate metabolism is the preferential use of fatty acid (FA) oxidation, but in order to adapt to the consumption of more ATP, energy metabolism transfers to glucose metabolism to produce more ATP. This increased glucose metabolism, however, results in the accumulation of pyruvate and lactate produced by glycolysis, which is harmful to the myocardium (Figure 2).

### Perhexiline

Perhexiline improves the energy production efficiency by transferring the substrate utilization from the free fatty acids (FFAs) to glucose and improves the symptoms, exercise capacity (VO_2_max), and function of heart in the patients with systolic heart failure caused by the ischemic and non-ischemic factors that are very effective (38). Perhexiline promotes the use of carbohydrates as the substrate for the myocardial energy by inhibiting carnitine palmitoyltransferase 1 (CPT1); meanwhile, CPT2 resulting in increased efficiency of the myocardial oxygen utilization (39). Perhexiline would be likely to induce an increase of at least 13% efficiency of the myocardial oxygen utilization (40). Perhexiline appears to exert the important anti-inflammatory (in part *via* nicotinamide adenine dinucleotide phosphate oxidase inhibition) and nitric oxide-potentiating effects that may occur independently of CPT inhibition (NCT00500552) (41).

### Mavacamten

During the period of myosin force production, there is an autoinhibited state, also referred to as a super-relaxed state. With certain myosin mutations, the HCM sarcomere spends lesser time in this state, resulting in the hyperactivation and excess utilization of ATP. The small molecule mavacamten stabilizes this inhibited state, effectively extend the time that myosin is inhibited. Mavacamten is specific for β-myosin heavy chain. Many studies in the mouse HCM models have pointed out that the early treatment of phenotype-negative HCM mice can prevent HCM hypertrophy and other features (42). Administration to the HCM mice reduced the hypertrophic phenotype and reduced the expression of the fibrotic genes (43). The gradient of 8 out of 21 participating patients had significantly reduced LVOT to <30 mm Hg. It also resulted in the reduced serum N-terminal pro B-type natriuretic peptide (NT-proBNP) levels in patients with HCM. This is the biomarker associated with increased wall stress and myocardial injury (NCT03470545) (43). The trial was designed to evaluate the dose of mavacamten in the non-obstructive HCM. This study found that the mavacamten treatment group had no significant toxicity compared to the placebo group, proving that the drug was well-tolerated (NCT03442764) (44).

### Omecamtiv Mecarbil

Omecamtiv mecarbil (OM) is being tested in treating hypercontractility by accelerating ATP generation, thus increasing myosin head binding to actin, resulting in an enhanced force-producing situation (45). The effects of OM are dependent on the intracellular Ca^2+^ levels (46). OM has shown promising clinical practical values, progressing to phase III trials (NCT02929329). The summarized metabolic therapy can be seen in Table 3.

**Table 3:** Table for metabolic therapy.

**Novel therapies**	**Targets**	**Mechanisms**
Perhexiline	CPT1/2	shifting LCFA→ glucose
Mavacamten	β-myosin heavy chain	stabilizes the super relaxed state
Omecamtiv mecarbil	Ca^2+^	accelerating ATP generation

## Cardiac Gene Therapy

In the past decades, gene therapy got tremendous development in the field of HCM. From the current evidence, gene therapy seems a very promising treatment in HCM caused by the mutations in the genes that encode the sarcomeric proteins.

The key problem for gene therapy is the effective and safe delivery of the gene therapy drugs into the body of the patient. It has been shown that the adeno-associated virus serotype 9 (AAV9) is a very promising candidate for cardiac gene transfer after systemic delivery in mouse and large animal HCM models (47). The SERCA2a gene therapy phase II trial also showed a very exciting result of the safety and feasibility of AAV1-mediated gene transfer (48). However, this investigation has not shown significant positive outcomes in the treated patients (49, 50). The defect of AAV-mediated gene therapy is that the human body easily generates neutralizing antibodies against AAV. These neutralizing antibodies seriously impact the outcomes of gene therapy; another question is an increased readministration rate. This could be avoided by the pharmacological modulation of the immune response and/or use of another AAV serotype (51, 52).

Fortunately, the difficulty of delivering the gene therapy medicinal product into the body of the patient has been resolved to some extent. Various methods were developed to suppress the expression of the genetic defects on the DNA or RNA levels as well as genome editing (42, 53–58), exon skipping (59, 60), allele-specific silencing, (61–63) spliceosome-mediated RNA trans-splicing (61–64), and gene replacement therapy (49, 65, 66). Due to the advancement in genome modification technologies, antisense oligonucleotides, clustered regularly interspaced short palindromic repeats (CRISPR)/Cas9, wild-type complementary DNA (cDNA) (wild-type MYBPC3 cDNA) sequences, and RNA interference molecules are clustered regularly interspaced. Specific editing that promotes the genetic mutations of an individual may lead to the individual-based pharmacological approaches in HCM. The summarized gene therapy can be seen in Table 4.

**Table 4:** Table for the gene therapy.

**Method**	**Medicinal product**	**Targeted**	**Results**	**Research status**
Genome editing	CRISPR/Cas9	mutated gene	repaired by homology-directed repair with a repair template	genetic correction in HCM hiPSC1-3 (55–57), and correct HCM caused by a GAGT-deletion in exon 16 of the MYBPC3 gene (58).
Exonskipping	antisense oligonucleotide	exonic splicing enhancer sequences of an inframe mutated exon	preventing binding of proteins involved in the splicing process	in newborn mice abolished cardiac dysfunction and prevented the development of leftventricularhypertrophy (59).
	CRISPR/Cas9	mutated DNA sequence	Permanently cut in-frame the mutated exon.	
Allele-specific silencing	specific RNA interferene molecule	mutant mRNA	knocked-down mutant mRNA	eliminate the mutant allele and delay the progression of cardiomyopathy in Myh6-targeted knock-in mice (63).
RNA trans-splicing	specific RNA interferene molecule	pre-mRNA	competes with cis-splicing	successful 5′trans-splicing in the context of HCM in cardiomyocytes and *in vivo* in Mybpc3-targeted knock-in mice and hiPSC (61, 62, 64).
Gene replacement	full-length cDNA	mutated DNA	functional full-length protein	in Mybpc3-targeted knock-in mice/hiPSCs, which were retrieved from an HCM patient carrying a truncating MYBPC3 muta -tion (49, 65, 66).

## Future Directions

Since there are many promising treatments for HCM, it is still a complex disease that requires further study based on pathophysiology and genetics. It is necessary to further study the mechanism of gene mutations and the secondary events caused by HCM. Thus, we need to develop new therapies based on gene editing or molecular regulatory pathways. Meanwhile, a large amount of basic medical research on the pathogenesis and treatment of HCM needs to be further transformed into clinical application. In conclusion, HCM is the main hereditary disease of the heart and the sarcomeric protein gene mutation is the most common cause of HCM. HCM is hereditary cardiomyopathy. Continued study and improved understanding of the genetic mediators of HCM will help to guide the development of effective targeted therapies, small molecules that can target the key molecular pathways or events in the heart to prevent the natural course of HCM. Increasing the treatment options for HCM may block the progression of the HCM disease, but it is not possible to completely correct the mutant gene and there are still genetic risks. Finally, a better understanding of the structural and metabolic disorders caused by the gene mutations is very helpful for developing the new therapies of HCM.

## Author Contributions

YG: conception. MA and ZC: administrative support. HQ: study design. TF: collection and assembly of data. JH: data analysis and drawing. All authors: manuscript writing and final approval of manuscript.

## Funding

This work was supported by the 1.3.5 Project for Disciplines of Excellence, West China Hospital, Sichuan University (grant number: ZY 2017306) and Sichuan Provincial Scientific Grant: The effect and mechanism of HIPPO-YAP pathway on pulmonary artery remodeling in left heart disease-related pulmonary hypertension (2021YFS0247).

## Conflict of Interest

The authors declare that the research was conducted in the absence of any commercial or financial relationships that could be construed as a potential conflict of interest.

## Publisher's Note

All claims expressed in this article are solely those of the authors and do not necessarily represent those of their affiliated organizations, or those of the publisher, the editors and the reviewers. Any product that may be evaluated in this article, or claim that may be made by its manufacturer, is not guaranteed or endorsed by the publisher.
